# Validity of Four Commercial Bioelectrical Impedance Scales in Measuring Body Fat among Chinese Children and Adolescents

**DOI:** 10.1155/2015/614858

**Published:** 2015-06-08

**Authors:** Lin Wang, Stanley Sai-chuen Hui

**Affiliations:** ^1^Center for Sports Medicine and Rehabilitation, Shanghai University of Sport, Shanghai 200438, China; ^2^Department of Sports Science and Physical Education, The Chinese University of Hong Kong, Hong Kong

## Abstract

The aim of the study is to examine the validity in predicting body fat percentage (%BF) of different bioelectrical impedance (BIA) devices among Chinese children and adolescents. A total of 255 Chinese children and adolescents aged 9–19 years old participated in the study. %BF was assessed by BIA scales, namely, Biodynamics-310 (Model A), Tanita TBF-543 (Model B), Tanita BC-545 (Model C), and InBody 520 (Model D). Dual-energy X-ray absorptiometry (DXA) was used as the criterion measurement. Lin's concordance correlation coefficients of estimated %BF between Model A, Model B, Model C, and DXA showed poor agreements for both genders. Moderate agreements for %BF were found between DXA and Model D measurements. In boys, differences in %BF were found between DXA and Model B and Model C. No significant %BF differences were found between Model A, Model D, and DXA. However, the two BIA analyzers showed a significant positive correlation between the bias and average %BF between BIA and DXA. In girls, differences in %BF were observed between Model B, Model C, Model D, and DXA. Model A and DXA showed no significant differences of %BF; however, the bias and the average %BF between the BIA and DXA had a significant positive correlation. Using embedded equations in BIA devices should be validated in assessing the %BF of Chinese children and adolescents.

## 1. Introduction

The World Health Organization has acknowledged that childhood obesity is one of the most serious public health challenges of the 21st century [1]. The International Obesity Task Force (IOTF) reported that 1 in 10 children aged 5–17 years is overweight, and 30–45 million children aged 5–17 years (accounting for 2-3% of the global population of children) are obese [2]. The prevalence of overweight and obesity has continued to increase at alarming rates in China, particularly among children and adolescents [3]. From 1982 to 2000, the prevalence of overweight and obesity among children and adolescents increased from 1.4% to 5.9% for boys and from 1.4% to 4.5% for girls [4].

Obesity is generally defined as abnormal or excessive fat accumulation that presents health risks [1]. Childhood obesity has significant adverse effects on health, including cardiovascular disease, type 2 diabetes, hypertension, stroke, and certain types of cancer [5]. In addition to physiologic risks, psychological morbidity is likely to be the most widespread health impact during childhood [6]. The measurement of body fat is central to many aspects of childhood obesity research [7, 8]. It is important to have valid and reliable tools to assess body fat. Devices that accurately assess body fat percentage (%BF) can be used to determine an individual's body composition or weight loss over a certain period.

Many laboratory methods have been developed to measure body composition. Such techniques include underwater weighing, air-displacement plethysmography, deuterium dilution, dual-energy X-ray absorptiometry (DXA), computerized tomography (CT), and magnetic resonance imaging (MRI) [9]. These methods have provided researchers with opportunities to measure human body fat precisely [10]. DXA can provide acceptable accuracy and reliability in measuring body composition among children [11]. This has been justified by its successful validation against the multicomponent model for children [12]; therefore, DXA is popularly used as a method of measuring children's body composition [13]. However, because of its inaccessibility, the high cost of equipment, and exposure to low-dose radiation, the use of DXA is limited in the field setting. Thus, simple methods such as bioelectrical impedance analysis (BIA) and skinfold thickness (SKF) measurement are used in field studies [9].

Compared with methods of measuring body composition, BIA is relatively simple, quick, and noninvasive. It gives reliable measurements of body composition with small intra- and interobserver variability [14]. BIA does not require exposure to radioactivity or submersion in water, which is why it is regarded as a practical measure of body composition, especially for field studies involving children. Because of its advantages, many BIA consumer devices are popular with health professionals and the general public for the assessment and monitoring of body fat. These devices are characterized by differences in electrodes (number, type, and placement), electric current frequency, and body position at measurement [15]. Single-frequency BIA devices with four electrodes, such as a traditional hand-to-foot BIA machine using adhering electrodes and a foot-to-foot BIA machine with four metal electrodes, have been used widely to assess body composition [15]. Multifrequency hand-to-foot BIA machines with eight electrodes have been developed for assessing segmental and total body compositions [16]. Multifrequency BIA was suggested to be a more accurate method for estimating the body composition of adults [17]. Segmental multiple-frequency BIA devices employ a current pathway that bypasses the arms, legs, and trunk using current different frequencies [18]. The most appropriate combination of frequencies and multivariate methods of using multi-frequency impedance values in estimating body composition are just now being explored; therefore, bioelectrical impedance spectroscopy (BIS) devices are designed to scan a wide range of frequencies; this approach provides estimates of extracellular water, intracellular water, and total body water [19].

Foot-to-foot BIA consumer devices are widely used to assess body composition among children and adolescents. Some studies have investigated the accuracy of single-frequency foot-to-foot BIA devices in assessing body fat. Contradictory results were found between foot-to-foot BIA and the reference methods in assessing body composition among children and adolescents, with some studies showing an acceptable level of accuracy [20–23] and others reporting a poor level of accuracy [24–29]. Although multifrequency BIA was suggested to be a more accurate method for estimating adults' body composition [17], its accuracy in evaluating the body composition of children and adolescents is doubtful. Some studies considered multifrequency BIA as an accurate way to measure body fat in children and adolescents [30, 31], whereas others found poor accuracy between multifrequency BIA and criterion methods [10, 32]. These discrepancies may be associated with differences among BIA consumer devices and ethnic differences in body composition. Previous studies showed that East Asian and South Asian children and adolescents tend to have more body fat than Caucasians at an equivalent BMI [33–35]. To date, only two studies have been conducted to investigate the accuracy of foot-to-foot BIA consumer devices for the measurement of body fat percentage in Chinese children and adolescents against the DXA measurement [21, 23]. A good correlation of body fat percentage and acceptable agreement between BIA measurement and DXA measurement were identified. However, these studies were limited by a small sample size in a wide age range. Only single-frequency foot-to-foot BIA devices were tested in these studies. The accuracy of different portable BIA consumer devices in assessing body fat among Chinese children and adolescents remains questionable.

To keep track of childhood obesity levels effectively, it is necessary to evaluate the obesity level of children in China using objective and practical measurement methods. BIA consumer devices provide a cheap and easy way to measure the body composition of Chinese children and adolescents. However, no study has assessed the validity of different portable BIA devices in relation to a preferred criterion method. To achieve this, the accuracy of BIA consumer devices in predicting body fat among Chinese children and adolescents should be determined. Therefore, the aim of the present study is to examine the validity in measuring body fat.

## 2. Material and Methods

### 2.1. Participants

A total of 255 healthy Chinese children and adolescents aged 9–19 years were recruited from schools in Shanghai, China. Of the total, 127 were boys and 128 were girls. A stratified sampling method was used to recruit a heterogeneous sample covering a wide range of ages and body compositions according to age and gender specific BMI distributions among Chinese children. The stratified sampling method was used in our previous study. Written informed consent was obtained from each participant and his or her parents, and the study was approved by the Local Ethics Committee.

### 2.2. Anthropometrics Measurement

Body weight and height were measured with minimal clothing and bare feet. Body height was measured to the nearest 0.5 cm using a fixed stadiometer (Holtain, Crymych, Dyfed, United Kingdom). Body weight was measured to the nearest 0.1 kg using a standard scale (Tanita TBF-543, Tanita, Tokyo, Japan). A trained investigator performed all the measurements.

### 2.3. Measurement of Body Composition

#### 2.3.1. DXA Measurement

Dual-energy X-ray absorptiometry (GE Lunar Prodigy, software version 10.51.006, GE Healthcare, Madison, USA) was used as the criterion measurement for body composition in terms of fat mass (FM), lean body mass (LTM), and bone mineral contents (BMC). Each participant was scanned in the supine position using X-ray at two energy sources (40 keV and 70 keV) in fast mode. A series of transverse scans were done from head to toe at one-centimeter intervals. The time spent was about five minutes depending on the participant's height. A trained investigator completed all the measurements of the DXA scan.

#### 2.3.2. BIA Measurements

BIA measurements were undertaken at least two hours after breakfast and with an empty bladder. Girls having their menses avoided the tests. In the current study, body composition was assessed by four BIA scales, namely, Biodynamics-310 (Model A, Biodynamics Corp., Seattle, USA), Tanita TBF-543 (Model B, Tanita Corp., Tokyo, Japan), Tanita BC-545 (Model C, Tanita Corp., Tokyo, Japan), and InBody 520 (Model D, Biospace Co., Ltd., Seoul, Korea). Manufacturers' equations were used to predict %BF (using all scales) and fat-free mass (using Model A and Model D). Model A was a single-frequency traditional hand-to-foot BIA machine using adhering electrodes. The measurement was carried out with the participants lying supine on a couch. The arms were separated from the trunk by about 30°; the legs were abducted and separated by about 45°. The skin of the right hand and foot was cleaned with an alcohol pad before the electrodes were placed. Body composition was measured at 50 kHz with a tetrapolar arrangement of standard electrodes (Red Dot 2330, 3M Healthcare, Saint Paul, USA). Two electrodes were placed on the right ankle with one (the source electrode) just proximal to the third metatarsophalangeal joint and the other (the sensing electrode) on the anterior ankle between the medial and the lateral malleolus. Two electrodes were placed on the right wrist with one (the source electrode) just proximal to the third metacarpophalangeal joint and the other (the sensing electrode) on the posterior wrist between the styloid processes of the radius and the ulna. Model B was a bipolar single-frequency (50 kHz) foot-to-foot instrument. Participants were instructed to stand barefoot with heel and forefoot placed on the four metal electrodes. Model C was a dual-frequency (50 kHz and 6.25 kHz) and Model D was a multifrequency (5 kHz, 50 kHz, and 500 kHz) BIA device with eight electrodes in a tetrapolar arrangement. The measurements required the participants to stand barefoot on metal electrodes while grasping a pair of electrodes fixed on a handle with arms extended in front of the chest. All BIA measurements were completed by a trained investigator according to the device manufacturers' instructions.

### 2.4. Statistical Analysis

Descriptive statistics were reported as means ± standard deviations (SD). An independent* t*-test was employed to determine the differences in the participants' physical characteristics, DXA measurements, and BIA measurements between boys and girls. The 5% level was chosen for statistical significance. Lin's concordance correlation coefficient (*rc*) was used to measure the bivariate relationship of %BF and FFM obtained from DXA with those obtained from BIA measurements. McBride suggests the following descriptive scale for values of the concordance correlation coefficient (for continuous variables): <0.90 poor, 0.90–0.95 moderate, 0.95–0.99 substantial, and >0.99 almost perfect [36]. Differences in body composition between those assessed by BIA methods and DXA measures were examined using a paired* t*-test. Bland-Altman analysis was applied to determine the agreement between BIA and DXA measurements [37]. The agreement between methods is represented by the bias, estimated by the mean difference, and the SD of the differences. Therefore, the 95% limits of agreement were calculated as the mean difference ±1.96 SD of the differences between methods. The pure error in %BF between each BIA device with DXA measurement was assessed using the total error (TE) as follows:

(1) $$\begin{array}{c} \text{TE}=\sqrt{\sum \frac{{\left(Y-Y^{\prime }\right)}^{2}}{N}}, \end{array}$$

where *Y* = observed values, *Y*′ = predicted values, and *N* = the number of participants in the sample. These statistical analyses were performed separately on boys and girls.

## 3. Results

### 3.1. Characteristics of Participants

A total of 255 (128 girls and 127 boys) Chinese children and adolescents participated in the study.  Table 1 presents their physical characteristics and body compartments (FM, LTM, FFM, BMC, and %BF). No significant differences in mean age were observed between genders. The mean body weight, height, and BMI of boys were higher than those of girls. The mean of the individual body compartments was significantly different between boys and girls (Table 1). 80.3% of boys and 82.8% of girls had entered puberty (pubertal stage ≥2 for pubic hair or breasts (girls)/genitalia (boys)).

Table 2 shows Lin's concordance correlation coefficients between parameters of body composition assessed by different BIA devices and DXA methods. In boys, the FFM determined by all BIA devices showed substantial agreements with that determined by DXA (*rc* values within 0.951–0.979). In girls, the FFM determined by InBody 520 showed substantial agreement with that determined by DXA (*rc* = 0.957); moderate agreements for FFM were found on Biodynamics-310 (*rc* = 0.909) and Tanita BC-545 (*rc* = 0.910), and poor agreement for FFM was found on Tanita TBF 543 (*rc* = 0.897). Lin's concordance correlation coefficients of estimated %BF between Biodynamics-310, Tanita TBF-543, Tanita BC-545, and DXA showed poor agreements (*rc* values within 0.747–0.863) for both genders. Moderate agreements for %BF were found between DXA and InBody 520 measurements (*rc* = 0.926 in boys and 0.912 in girls).

In boys, significant differences in %BF were found between DXA and Model B and Model C (paired* t*-test, *p* < 0.05). The mean bias of %BF for Model B was 1.15% (SD = 5.95%) with 95% limits of agreement −10.50–12.81%. Model C underestimates %BF; the mean bias was −1.67% (SD = 4.8%) with 95% limits of agreement −11.08–7.74%. However, no significant %BF differences were found between Model A, Model D, and DXA. Model A and Model D BIA analyzers showed a significant positive correlation between the bias and average %BF between BIA and DXA, indicating that %BF was underestimated at low values and overestimated at high values in the present sample (Table 3).

In girls, significant differences in %BF were observed between Model B, Model C, Model D, and DXA. %BF was underestimated by the three BIA devices. The mean biases were −2.63% for Model B (SD = 3.23%, 95% limits of agreement −8.96–3.70%), −1.47% for Model D (SD = 2.99%, 95% limits of agreement −7.32–4.40%), and −2.05% for Model C (SD = 3.21%, 95% limits of agreement −8.34–4.24%). Model A and DXA showed no significant differences of %BF; however, the bias and the average %BF between BIA and DXA had a significant positive correlation, indicating that the %BF was underestimated at low values and overestimated at high values in the present sample (Table 3). Model A, Model C, and Model D overestimated the FFM in boys. All BIA devices overestimated the FFM in girls (Table 3).

## 4. Discussion

Chinese children and adolescents are at increased risk for obesity and associated health risks. Determining weight status through measurement of body fat percentage is important in diagnosing childhood obesity. Several methods, such as DXA, Bod Pod, and underwater weighing, can provide accurate body fat measurement. However, these measurements are often inaccessible to the larger scale population survey. The search for an accurate diagnostic instrument for measuring body fat that is practical and inexpensive is a challenge for researchers. In this sense, commercial BIA has become a popular method due to its ease of use and low cost [38]. Numerous different BIA commercial devices can measure body composition, but their suitability for Chinese children and adolescents is still unclear. The accuracy of BIA measurement may be influenced by several factors, such as body shape, hydration status, and ethnicity [9]. Previous studies have suggested that body composition from commercial BIA devices is derived from manufacturers' equations, which are often not validated for specific ethnic groups [38–40]. These discrepancies may be associated with differences among the devices and ethnic differences on body composition [10, 26]. We sought investigating the validity and diagnostics accuracy of several different BIA devices in assessing excessive body fat in Chinese children and adolescents. The findings of the current study may determine useful BIA devices for local health practitioners in assessing obese children.

Previous validation studies in children and adolescents have found that different BIA (foot-foot, hand-foot, and multiple frequencies) devices using manufacturers' equations tend to overestimate or underestimate %BF in relation to criteria measurement [24–28, 32]. Single-frequency foot-foot or hand-foot BIA devices generally tend to underestimate or overestimate %BF in children and adolescents. In the current study, although high correlations in body composition measurements between BIA devices and DXA were found, foot-foot (TBF-543) BIA device overestimated %BF in boys and underestimated %BF in girls in comparison with DXA measurement. Most of previous studies found that Tanita BIA devices underestimated %BF in both genders [24, 25, 27, 37], with which the findings of the current study was consistent for girls. However, in current boys, Tanita TBF-543 overestimated %BF (bias = 1.15 ± 5.59%) with negative correlation between bias and averaged %BF between BIA and DXA measurements. The discrepancies may be explained by different testing population, ethnicity, and use of Tanita BIA devices with different models. The current study uses a heterogeneous and relatively larger sample covering a wide range of ages and body compositions according to age and gender specific BMI distributions among Chinese children. To date, two studies were conducted to investigate the accuracy of foot-foot BIA consumer devices in measuring body fat percentage in Chinese children and adolescents compared with DXA measurement [21, 23]; however, these studies had several limitations. Lu et al. measured %BF of 64 Chinese obese children aged 10–17 years using Tanita-401, and no significant differences were found in %BF between foot-foot BIA and DXA [21]. Moreover, BIA overestimated the %BF in seriously obese children. Sung et al. found that BIA (Tanita TBF 401) slightly overestimated %BF in 7–18-year-old Chinese children (*n* = 49); however, the %BF bias did not reach statistical significance [23]. The two studies used smaller samples with a wider age range to investigate the validity of foot-foot measured %BF, which may significantly explain the different findings between the current and previous studies.

In addition, despite no mean bias was found for the %BF between the hand-foot BIA device (Biodynamics-310) and DXA measurement in both genders, the Bland-Altman analysis showed that the two BIA devices overestimated measurements at a low %BF and underestimated them at high %BF. Previous studies investigated the validity of hand-foot BIA devices in assessing %BF in children [22, 27, 28, 41]. Several studies had the same results with that of the present study [22, 41].

The eight-tactile electrodes with multiple frequencies BIA device could estimate body composition in adults more accurately than a single-frequency BIA device [17, 42]. However, contradictory findings were revealed on the validity of multiple frequencies BIA devices in measuring body composition in children and adolescents. Two studies did not show statistical significance in %BF between multiple frequencies BIA (InBody) and DXA measurements [30, 31]. However, Jensky-Squires et al. found that InBody 320 overestimated %BF in female adolescents from underwater weighing [10]. In two other studies, Prins et al. found that Tanita BC-418 overestimated %BF determined by deuterium dilution in African children [43]; Sluyter et al. found that the same BIA device underestimated %BF in a multiethnic group of adolescents. Moreover, the BIA device tended to underestimate measurements in lower %BF and overestimate them in higher %BF individuals [32]. In the current study, two multiple frequencies BIA devices (InBody 520 and Tanita BC-545) were also used to evaluate body composition. Tanita BC-545 underestimated %BF in both genders and InBody 520 underestimated %BF in girls. In boys, no bias was found in %BF between InBody 520 and DXA measurement, but a significant positive correlation was found between the bias and averaged %BF between BIA and DXA. InBody 520 thus overestimated %BF at high values and overestimated it at low values in boys. For Tanita multiple frequencies BIA devices, the results of the current and previous studies are consistent. The devices underestimated %BF in children and adolescents. For the InBody multiple BIA devices, the varying results may have been caused by different population and ethnicity.

Moreover, note that although the mean bias may be small, of higher relevance are the 95% limits of agreement, which seem to more accurately reflect the validity of measurement in an individual. Numerous studies found that the BIA has quite wide limits of agreement with criterion measurement of %BF [24, 26, 27, 41, 44]; therefore, BIA is not interchangeable with criterion measurement for body composition. As such is true even for Chinese children and adolescents, as in the current study, the 95% limits of agreement of all BIA devices showed wide ranges, in agreement with previous studies. Sung et al. found that the 95% limits of agreement in %BF were narrow (from −3.92 to 0.61%) between foot-foot BIA and DXA in 17 obese and 32 nonobese Chinese children aged 7–18 years [23]. The current study had larger 95% limits of agreement in the same ethnicity and similar age range of population. The discrepancy may be partly explained by different samples size, sampling method, and DXA instruments used (Hologic QDR-4500 and GE Lunar Prodigy). Previous studies suggested that if researchers intend to measure body composition in large-scale population surveys, then BIA measurement may provide an efficient alternative to laboratory measurement of body composition when accurate instruments are unavailable [45]. BIA measurement should be used with caution in evaluating or monitoring changes in individual body composition in healthy and clinical setting [38].

## 5. Conclusion

Using embedded equations in BIA devices should be validated in assessing the body composition of Chinese children and adolescents aged 9–19 years old. All BIA devices are not directly interchanged with DXA measurement in assessing individual %BF and assessing changes of body composition owing to the wide limits of agreement.

## Figures and Tables

**Table 1:** Physical characteristics and body compositions of the study population.

	Boys (*n* = 127)			Girls (*n* = 128)		
	Mean (SD)	Range		Mean (SD)	Range	
		Lower	Upper		Lower	Upper
Age (years)	13.8 (2.9)	9.0	19.5	13.7 (2.8)	9.0	19.4
Height (cm)	161.8 (14.0)	130.5	186.0	154.3 (9.8)	126.6	172.0
Weight (kg)	55.9 (17.0)	27.5	108.1	47.4 (11.3)	23.5	97.1
BMI (kg^2^/m)	21.0 (4.2)	13.6	37.1	19.7 (3.2)	13.3	34.8
LTM-DXA (kg)	41.0 (11.2)	21.0	65.3	31.0 (5.5)	18.7	54.3
BMC-DXA (kg)	2.0 (0.7)	0.9	3.8	1.7 (0.5)	0.8	2.7
FM-DXA (kg)	12.7 (9.5)	3.3	53.1	14.3 (6.8)	2.7	40.6
FFM-DXA (kg)	43.0 (11.8)	22.1	69.0	32.8 (5.9)	19.5	56.8
FFM-Model A (kg)	43.8 (12.7)	21.8	77.4	33.9 (6.5)	18.6	61.2
FFM-Model B (kg)	43.2 (11.7)	20.8	73.2	34.7 (5.5)	20.6	51.7
FFM-Model C (kg)	44.5 (12.0)	22.6	73.2	34.5 (5.5)	20.6	51.6
FFM-Model D (kg)	44.3 (12.2)	22.8	72.0	34.0 (5.9)	20.0	55.3
%BF-DXA (%)	20.8 (9.9)	6.1	49.1	27.9 (7.7)	11.8	46.5
%BF-Model A (%)	21.4 (7.0)	4.9	39.6	27.4 (6.0)	13.6	42.1
%BF-Model B (%)	21.9 (6.9)	10.1	45.3	25.3 (7.8)	9.3	49.4
%BF-Model C (%)	19.0 (9.0)	5.0	54.7	25.8 (7.7)	10.3	47.6
%BF-Model D (%)	20.2 (8.5)	6.2	47.3	26.3 (8.1)	6.4	44.8

Note: data are presented as mean (SD); BMI: body mass index; LTM-DXA: lean tissue mass measured by dual-energy X-ray absorptiometry; BMC-DXA: bone mineral content measured by dual-energy X-ray absorptiometry; FM-DXA: fat mass measured by dual-energy X-ray absorptiometry; %BF-DXA: body fat percentage measured by dual-energy X-ray absorptiometry; FFM-DXA: fat-free mass calculated as the sum of LTM and BMC; Model A: Biodynamics-310; Model B: Tanita TBF 543; Model C: Tanita BC-545; Model D: InBody 520.

**Table 2:** Lin's concordance correlation coefficients between parameters assessed by different BIA and DXA measurement.

		BIA devices			
		Model A	Model B	Model C	Model D
Boys (*n* = 127)	%BF-DXA	0.765	0.750	0.855	0.926
	FFM-DXA	0.953	0.953	0.951	0.979

Girls (*n* = 128)	%BF-DXA	0.747	0.863	0.881	0.912
	FFM-DXA	0.909	0.897	0.910	0.957

Note: BMI: body mass index; %BF-DXA: body fat percentage measured by dual-energy X-ray absorptiometry; FFM-DXA: fat-free mass measured by dual-energy X-ray absorptiometry.

Model A: Biodynamics-310; Model B: Tanita TBF 543; Model C: Tanita BC-545; Model D: InBody 520.

**Table 3:** Cross-validation of four BIA devices (embedded equations) for the perdition of %BF/FFM in Chinese children and adolescents.

Gender	BIA devices	%BF-DXA	%BF-BIA	FFM-DXA	FFM-BIA	*p* *t*-test	Bias (SD)	TE	*r*_*y*−*y*′,*y*_	95% limits of agreement
Boys (*n* = 127)	Model A	20.77%	21.34%	—	—	0.268	0.58 (5.86)%	5.86%	0.513^*∗∗*^	−10.96% to 12.12%
	Model B	20.77%	21.92%	—	—	0.031	1.15 (5.95)%	6.04%	0.528^*∗∗*^	−10.51% to 12.81%
	Model C	20.77%	19.04%	—	—	<0.001	−1.67 (4.80)%	5.06%	0.162	−11.08% to 7.74%
	Model D	20.77%	20.22%	—	—	0.246	−0.37 (3.51)%	3.51%	0.365^*∗∗*^	−7.25% to 6.51%
	Model A	—	—	43.02 kg	43.80 kg	0.019	0.78 (3.70) kg	3.77 kg	0.261^*∗∗*^	−6.47 kg to 8.03 kg
	Model B	—	—	43.02 kg	43.18 kg	0.608	0.16 (3.60) kg	3.60 kg	−0.027	−6.90 kg to 7.22 kg
	Model C	—	—	43.02 kg	44.50 kg	<0.001	1.54 (3.35) kg	3.60 kg	0.024	−5.03 kg to 8.11 kg
	Model D	—	—	43.02 kg	44.31 kg	<0.001	1.02 (2.17) kg	2.39 kg	0.197^*∗*^	−3.23 kg to 5.27 kg

Girls (*n* = 128)	Model A	27.85%	27.37%	—	—	0.266	−0.48 (4.88)%	4.88%	0.359^*∗∗*^	−10.04% to 9.08%
	Model B	27.85%	25.22%	—	—	<0.001	−2.63 (3.23)%	4.15%	−0.049	−8.96% to 3.70%
	Model C	27.85%	25.81%	—	—	<0.001	−2.05 (3.21)%	3.79%	0.012	−8.34% to 4.24%
	Model D	27.85%	26.34%	—	—	<0.001	−1.47 (2.99)%	3.31%	−0.147	−7.33% to 4.39%
	Model A	—	—	32.77 kg	33.89 kg	<0.001	1.11 (2.42) kg	2.66 kg	0.245^*∗∗*^	−3.63 kg to 5.85 kg
	Model B	—	—	32.77 kg	34.74 kg	<0.001	1.97 (1.77) kg	2.64 kg	−0.255^*∗∗*^	−1.50 kg to 5.44 kg
	Model C	—	—	32.77 kg	34.49 kg	<0.001	1.72 (1.77) kg	2.47 kg	−0.219^*∗∗*^	−1.75 kg to 5.19 kg
	Model D	—	—	32.77 kg	34.03 kg	<0.001	1.25 (1.22) kg	1.68 kg	−0.022	−1.14 kg to 3.64 kg

Note: %BF: body fat percentage; FFM: fat-free mass; DXA: dual-energy X-ray absorptiometry; BIA: bioelectrical impedance analysis.

Bias: value obtained from BIA measurement minus DXA measurement; SD: standard deviation.

*r*_*y*−*y*′,*y*_, Pearson's correlation coefficient for the relationship between averaged %BF/FFM [(value of DXA + value of BIA)/2] and the bias.

^*∗*^ *p* < 0.05; ^*∗∗*^ *p* < 0.01.

Model A: Biodynamics-310; Model B: Tanita TBF 543; Model C: Tanita BC-545; Model D: Inbody 520.
